# 1-Methyltryptophan treatment ameliorates high-fat diet-induced depression in mice through reversing changes in perineuronal nets

**DOI:** 10.1038/s41398-024-02938-4

**Published:** 2024-05-30

**Authors:** Juntao Hu, Shanshan Zhang, Haoran Wu, Leilei Wang, Yuwen Zhang, Hongyang Gao, Meihui Li, Hong Ren, Honglei Xiao, Kun Guo, Wensheng Li, Qiong Liu

**Affiliations:** 1 Department of Anatomy, Histology and Embryology, School of Basic Medical Sciences, Fudan University, Shanghai, China; 2 Institute of Science and Technology for Brain-Inspired Intelligence, Fudan University, Shanghai, China; 3 Electron Microscopy Core Laboratory, School of Basic Medical Science, Fudan University, Shanghai, China; 4 Key Laboratory of Carcinogenesis and Cancer Invasion, Liver Cancer Institute, Zhongshan Hospital, Fudan University, Shanghai, China; 5 Cancer Research Center, Institute of Biomedical Science, Fudan University, Shanghai, China; 6 Key Laboratory of Medical Imaging Computing and Computer Assisted Intervention of Shanghai, Shanghai, China; 7 State Key Laboratory of Medical Neurobiology and MOE Frontiers Center for Brain Science, Institutes of Brain Science, Fudan University, Shanghai, China

**Keywords:** Epigenetics and behaviour, Physiology

## Abstract

Depression and obesity are prevalent disorders with significant public health implications. In this study, we used a high-fat diet (HFD)-induced obese mouse model to investigate the mechanism underlying HFD-induced depression-like behaviors. HFD-induced obese mice exhibited depression-like behaviors and a reduction in hippocampus volume, which were reversed by treatment with an indoleamine 2,3-dioxygenase (IDO) inhibitor 1-methyltryptophan (1-MT). Interestingly, no changes in IDO levels were observed post-1-MT treatment, suggesting that other mechanisms may be involved in the anti-depressive effect of 1-MT. We further conducted RNA sequencing analysis to clarify the potential underlying mechanism of the anti-depressive effect of 1-MT in HFD-induced depressive mice and found a significant enrichment of shared differential genes in the extracellular matrix (ECM) organization pathway between the 1-MT-treated and untreated HFD-induced depressive mice. Therefore, we hypothesized that changes in ECM play a crucial role in the anti-depressive effect of 1-MT. To this end, we investigated perineuronal nets (PNNs), which are ECM assemblies that preferentially ensheath parvalbumin (PV)-positive interneurons and are involved in many abnormalities. We found that HFD is associated with excessive accumulation of PV-positive neurons and upregulation of PNNs, affecting synaptic transmission in PV-positive neurons and leading to glutamate-gamma-aminobutyric acid imbalances in the hippocampus. The 1-MT effectively reversed these changes, highlighting a PNN-related mechanism by which 1-MT exerts its anti-depressive effect.

## Introduction

The relationship between diet-induced obesity and mental illness has long been a subject of interest [1]. Obesity is currently one of the most critical public health burdens, affecting approximately 13% of the adult population globally. Well-established contributors to the rise in obesity incidence include western diet [2] and changes in labor patterns [3]. Notably, a higher prevalence of depression is reported among obese individuals, indicating a potential correlation between obesity and depression [4, 5]. Excessive consumption of a high-fat diet (HFD) is a major cause of obesity and has been linked to the development of mood disorders [6]. However, the underlying mechanisms of HFD-induced depression remain unclear.

Diet has been shown to directly modulate brain function [4, 5, 7]. Serotonin, a crucial neurotransmitter implicated in depression development, is affected by dietary fat intake, which interferes with its synthesis [5]. At present, commonly prescribed antidepressants primarily focus on regulating serotonin and other neurotransmitters that are involved in brain functions related to mood and behavior regulation, including norepinephrine and dopamine. However, in many cases, these antidepressants fail to adequately alleviate symptoms. The 5-hydroxytryptamine (5-HT) is a key neurotransmitter in the central nervous system (CNS). Increased synaptic 5-HT availability in the CNS is associated with improved mood and reduced anxiety, serving as the basis of several widely used antidepressants [8]. 5-HT is also an important metabolic hormone, contributing to the regulation of glucose homeostasis and obesity [9]. Tryptophan (Trp) is a precursor of 5-HT that can be metabolized through the methoxyindole and kynurenine (Kyn) pathways. The Kyn pathway, which takes up about 95% of the biologically available Trp, is controlled by the rate-limiting enzymes indoleamine 2,3-dioxygenase (IDO) and Trp 2,3-dioxygenase (TDO). IDO-mediated Trp degradation results in reduced 5-HT synthesis and increased production of Trp catabolic metabolites with key neurotoxic properties associated with depression [10–12]. In a Bacillus Calmette-Guerin-induced depression mouse model, inhibition of IDO by the pharmacologically competitive antagonist 1-methyltryptophan (1-MT) prevented the emergence of depression-like behaviors [13]. Moreover, 1-MT treatment has demonstrated antidepressant effects in streptozotocin-induced diabetic rats [13]. Therefore, IDO inhibition may be a potential therapeutic target for obesity, depression, and associated neuropsychiatric symptoms.

The hippocampus is a key brain region for emotion regulation and is highly vulnerable to metabolic dysfunction. It has been shown that 1-week exposure to HFD can lead to a notable increase in the expression of proinflammatory cytokines and the permeability of the blood-brain barrier, as well as memory and learning impairment, depressive-like behaviors, and synaptic changes. Later hippocampal alterations after 4 weeks of HFD consumption include mitochondrial dysfunction and astrocytic activation [14]. Furthermore, prolonged HFD intake for 12 weeks leads to hippocampal astrocyte dysfunction, downregulation of glial glutamate transporters, and induction of depression-like behaviors in mice [15]. However, the precise mechanism by which HFD induces these changes is still being investigated. One subject of attention is the perineuronal nets (PNNs), a form of extracellular matrix (ECM) predominantly localized to parvalbumin (PV)-expressing, gamma-aminobutyric acid (GABA)-releasing interneurons and is thought to influence their ability to fire [16–19]. PNNs have been implicated in various neurofunctions, such as neuroprotection, regulation of neural activity, and experience-related plasticity. PNN alterations have been observed in a variety of neurodevelopmental and neurodegenerative processes [20, 21]. For example, postnatal exposure to antidepressant selective serotonin reuptake inhibitors has been found to alter the trajectory of PNN development and local inhibitory circuit function in the hippocampus [22]. Growing evidence supports the role of diet in PNNs. Exposure to dietary fat has been associated with a decreased number of PNNs in the orbitofrontal cortex [23] and lower PNN intensity in the prefrontal cortex [24]. However, little is known about the diet-induced PNN changes in the hippocampus and their association with depression. Herein, we adopted an HFD-induced obesity mouse model to investigate the mechanism underlying HFD-induced depression-like behaviors and the impact of 1-MT treatment on such behaviors.

## Materials and methods

### Animals and dietary intervention

C57BL/6JRj male mice (8-week-old) were housed in four per cage in a constant-temperature room with a 12 h/12 h light/dark cycle and provided with free access to food and water. Following a 1-week habituation period, mice were randomly assigned to different groups to receive either a chow diet (CD, containing 10% fat calories) or an HFD (containing 60% fat calories) (D12492, research diets) for 23 weeks before behavior tests (*n* numbers: CD = 11, HFD = 10, HFD veh = 10, HFD 1-MT = 8). This study was conducted in compliance with the National Institutes of Health Guide for the Care and Use of Laboratory Animals. The protocol was approved by the Animal Ethics Committee of Shanghai Medical College of Fudan University. Due to the exploratory nature of this study, no formal power analysis or sample size estimation was conducted, and the sample size estimation was based on prior experience and literature [25].

### 1-MT treatment

1-MT (Sigma-Aldrich, 860646) was diluted in 0.1 M NaOH and then adjusted pH to 9.0 with 1 M HCl. The CD group received a solvent prepared with 1 M HCl and 0.1 M NaOH (pH = 9.0). Mice were subcutaneously injected with either the 1-MT solution (50 mg/kg) or solvent (equal volume to the 1-MT solution) twice a day for 5 weeks.

### Intraperitoneal glucose tolerance test (IPGTT)

IPGTT was performed after mice had fasted for 16 h. After the determination of basal blood glucose levels, each animal received an intraperitoneal injection of 2 mg/g glucose (G5767, Sigma), and blood glucose levels were measured at 15 min, 30 min, 60 min, and 120 min after glucose administration using a glucometer (Bayer HealthCare). The area under the curve of blood glucose levels throughout the experiment was calculated from the blood glucose level-time curve as an indicator to assess the glucose tolerance of mice.

### Behavioral tests

Behavioral tests, including the tail suspension test (TST), forced swimming test (FST), open field test (OFT), elevated zero maze (EZM) test, and sucrose preference test (SPT), were performed as previously described. Details can be found in the supplemental material. All behavioral tests were conducted using double-blinded measurements.

### Immunohistochemistry

Immunohistochemistry of mouse brain tissue was conducted as previously described. Briefly, mice were anesthetized, and fixed brain tissue was sliced into sections at 40 μm thickness. Slides were then stained with primary and secondary antibodies, and images were obtained using a Zeiss confocal microscope (LSM 700). Details can be found in the supplemental material.

### RNA sequencing and pathway enrichment analyses

Genergy Bio was commissioned to perform RNA extraction and transcriptome sequencing. Details can be found in the supplemental material. Differential transcripts between the two groups were screened based on a differential expression range of |log2FC| ≥ 1 and *P* value ≤ 0.05. Pathway analysis was performed on 130 shared differentially expressed genes (DEGs). The top Gene Ontology processes were enriched by the Metascape web-based platform [26]. The data that support the findings of this study are available in the NCBI repository database, reference number PRJNA1095641.

### Quantitative real-time polymerase chain reaction

Total RNA from mouse hippocampal tissue was extracted using TRIzol (R401-01, Vazyme). Extracted total RNA was reverse transcribed to cDNA using the PrimeScript RT Master Mix (RR036A, TaKaRa). A SYBR Green kit (Q711-02, Vazyme) was used for polymerase chain reaction quantification on the QuantStudio 5 (Applied Biosystems). The cycle threshold values of the target mRNA were collected and its expression level was normalized to β-actin. The relative expression of mRNAs was calculated using the 2-ΔΔCT method, and the results are presented as fold changes of the control group. The primers used in the present work were as follows: Lgals3 forward: GGAGAGGGAATGATGTTGCCT, reverse: TCCTGCTTCGTGTTACACACA; Col6a5 forward: CCAAACATGACACGGATCATCA, reverse: GGAACTGTCTTATCAACGTGGT; GAPDH forward: TGTGTCCGTCGTGGATCTGA; reverse: TTGCTGTTGAAGTCGCAGGAG.

### Magnetic resonance imaging scans and 1H-magnetic resonance spectroscopy

MRI scans were conducted using a small animal 11.7 T Bruker Biospec high-field MRI system. Details can be found in the supplemental material.

### High-performance liquid chromatography (HPLC) analysis

The levels of 5-HT, Trp, and Kyn in the hippocampus were determined by HPLC. The hippocampus was deproteinized in 0.2 N perchloric acid solution at 4 °C, then centrifuged at 12,000 rpm for 20 min, and the supernatant was analyzed. Chromatographic condition: chromatographic column (Welch Ultimate XB-C18 150 × 4.6 mm, 5 μm), flow rate (0.8 mL/min), water phase (0.1% formic acid/water), organic phase (0.1% formic acid/acetonitrile), and the column temperature box temperature was 40 °C. Chromatogram acquisition and integration of each analyte were processed using the Xcalibur software (Thermo Fisher).

### Transmission electron microscopy (TEM)

After mice were executed, approximately 1 mm^3^ of hippocampal tissue was promptly isolated and fixed in 2.5% glutaraldehyde for 16 h at 4 °C, followed by 1% osmium fixation for 1 h. The fixed sample was then dehydrated using a graded ethanol series and embedded in resin. Embedded sample blocks were trimmed and cut using an ultramicrotome, and the sections were then placed on a 200-slot grid coated with polyvinyl alcohol ester and imaged under a transmission electron microscope (CM120, Philips, London, UK). For TEM experiments, two mice were taken from each group, and three fields of view of the hippocampus were taken from each mouse.

### Data analysis

Prism software (version 8.30, GraphPad) was used for statistical analysis. One-way analysis of variance (ANOVA) in combination with Tukey’s multiple comparisons test was used for multiple comparisons, and two-way ANOVA was used for the comparison of intensity shifts. *P* < 0.05 was considered statistically significant. All data are presented as mean ± standard error of the mean.

## Results

### HFD induces depression-like behaviors in mice, which can be reversed by 1-MT treatment

**Fig. 1:**
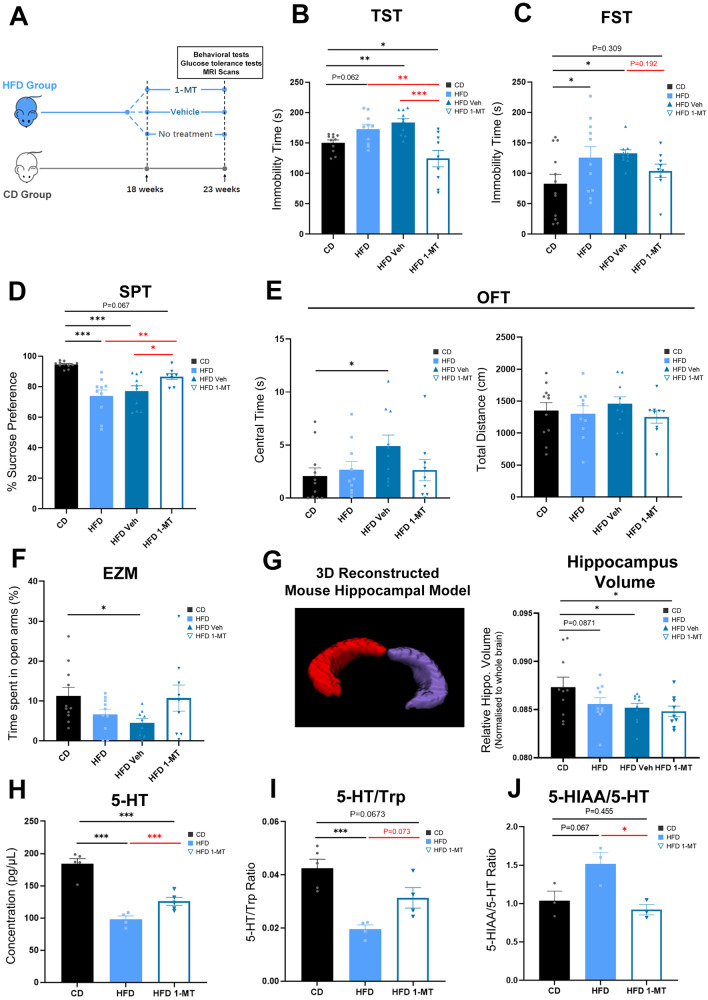
HFD-induced obesity mouse model and associated behavioral phenotypes. **A** Experimental design. The HFD-induced obesity mouse model was established by feeding 8-week-old male wildtype mice either HFD or standard CD for 18 weeks, followed by treatment with 1-MT, vehicle, or no treatment for 5 weeks. **B**, **C** Results of depressive behavior tests (TST and FST). **D** Results of the pleasure deficiency test (SPT). **E**, **F** Results of anxiety behavior tests (OFT and EZM). **G** 3D reconstructed mouse hippocampal model based on MRI and the quantification of hippocampal volume in mice. **H**–**J** Hippocampus 5-HT level, 5-HT/Trp ratio, and 5-HIAA/5-HT ratio post-1-MT treatment. Error bars represent the standard error of the mean. *P < 0.05, **P < 0.01, ***P < 0.001, as determined by the one-way ANOVA test in combination with Tukey’s multiple comparisons test. *N* numbers are indicated in each graph. EZM elevated zero mazes, 5-HIAA 5-hydroxyindoleacetic acid, 5-HT 5-hydroxytryptamine, CD chow diet, FST forced swimming test, HFD high-fat diet, OFT open field test, SPT sucrose preference test, Trp Tryptophan, TST tail suspension test.

To investigate the comorbidity of obesity and associated behavioral phenotypes, we established an HFD-induced obesity mouse model by feeding 8-week-old male wildtype mice either HFD or standard CD for 23 weeks (Fig. 1A). As expected, HFD-fed mice gained more body weight and had significantly higher WAT masses compared to CD-fed mice (Fig. S1A–D). Similarly, HFD-fed mice developed hyperglycemia and exhibited impaired glucose tolerance (Fig. S1E–G). In behavior assays for depression-like behaviors, HFD-fed mice had mildly prolonged immobility in the TST and increased immobility time in the FST (Fig. 1B, C). Also, HFD-fed mice exhibited reduced sucrose preference, indicating anhedonia (Fig. 1D). No significant changes were observed in anxiety-like behaviors in HFD-fed mice compared to the CD-fed mice, as determined by OPF and EZM tests (Fig. 1E, F). These results collectively demonstrate that prolonged consumption of HFD induces depression-like behavior in mice without significant alterations in anxiety-like behaviors.

Given the close association between hippocampal volume reduction and the onset of depression, we examined hippocampal volume by MRI. Our findings revealed that mice in the HFD group had a significant reduction in hippocampal volume with a normal whole brain volume compared with CD-fed mice (Figs. 1G and S1H). This suggests that HFD consumption can induce structural changes in the hippocampus.

The Trp metabolism pathway plays a crucial role in the pathogenesis of depression [27]. The two major downstream metabolism paths of Trp, the Kyn pathway and the serotonin pathway, are both involved in depression, though in different ways. We explored the involvement of the Trp metabolism pathway in the depression-like behavior observed in HFD-fed mice by assessing the levels of hippocampal Trp metabolites and enzymes. To our surprise, we found no significant effect of HFD on hippocampus Trp and Kyn levels or the Kyn/Trp ratio (Fig. S1J, K). However, we observed a significant reduction in the hippocampus 5-HT level and 5-HT/Trp ratio, as well as elevated blood 5-hydroxyindoleacetic acid (5-HIAA)/5-HT ratio in the HFD group, indicative of serotonin turnover (Fig. 1H–J). These observations align with literature stating that 5-HT is involved in mood regulation and the pathophysiology of depression, and that blood serotonin turnover is elevated in patients with depression [28, 29]. Our findings suggest that the serotonin pathway plays a more prominent role in HFD-induced depression-like behavior compared to the Kyn pathway.

To our surprise, 1-MT, an IDO inhibitor known to exert its antidepressant effect through competitively inhibiting IDO activity and reducing the production of Kyn pathway metabolites, showed a similar antidepressant effect in HFD-induced depression. Although the 5-week 1-MT treatment did not reduce body weight and only partially rescued eWAT masses in HFD-induced obese mice, 1-MT exhibited a therapeutic effect on impaired glucose tolerance (Fig. S1A–G). Although 1-MT did not show a rescuing effect on hippocampal volume in HFD-induced obese mice, behavioral tests indicated full or partial rescue of depression-like behavior in this group (Fig. 1B–D). Moreover, 1-MT improved the hippocampus 5-HT level and 5-HT/Trp ratio and restored the 5-HIAA/5-HT ratio (Fig. 1H–J). These results suggest that 1-MT treatment can reverse the depression-like behaviors observed in HFD-induced obese mice.

### 1-MT reverses the altered ECM-associated gene expression observed in HFD-induced depression

**Fig. 2:**
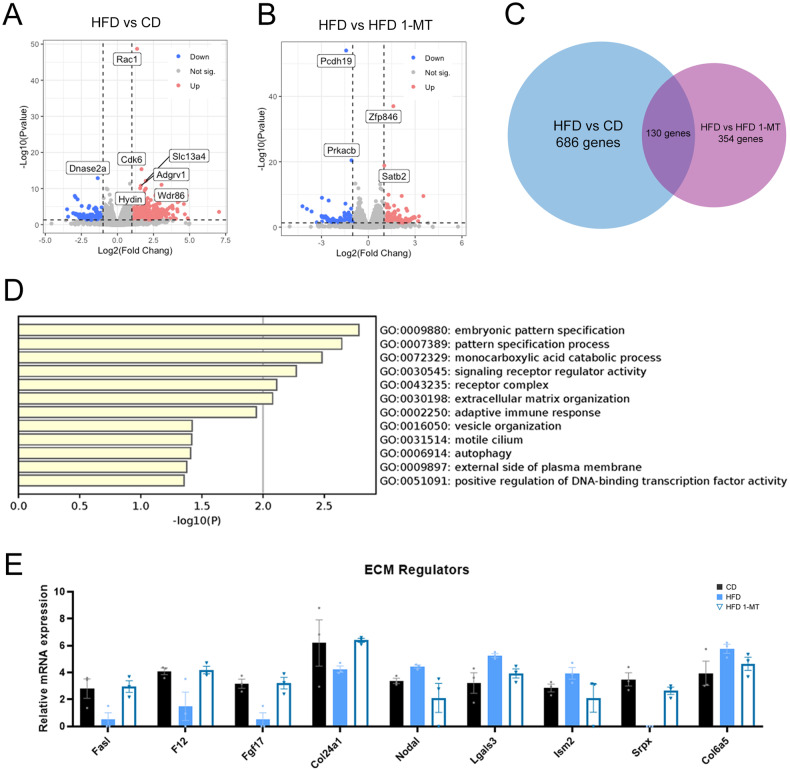
RNA sequencing analysis for exploring DEGs and signaling pathways in the hippocampal expressional profiles of mice in the CD group, HFD group (with and without 1-MT treatment). **A** DEGs between the CD and HFD groups. **B** DEGs between the HFD group and the HFD group treated with 1-MT. **C** Genes cross-referenced between the CD group, HFD group, and HFD group treated with 1-MT. **D** Pathway enrichment analysis of 130 shared differential genes. **E** Relative mRNA expression of the nine identified ECM regulators in the CD group, HFD group, and HFD group treated with 1-MT. Error bars represent the standard error of the mean. *N* = 3 for each group. 1-MT 1-methyltryptophan, CD chow diet, ECM extracellular matrix, HFD high-fat diet.

To investigate the molecular mechanism involved in the therapeutic effect of 1-MT in reversing such phenotype, we performed RNA sequencing analysis to explore DEGs and signaling pathways in the hippocampal expressional profiles of mice in the HFD and CD groups with and without 1-MT treatment. We identified 816 DEGs (554 upregulated, 262 downregulated) in the HFD group relative to the CD group (Fig. 2A), and 484 DEGs (227 upregulated, 207 downregulated) in the HFD + 1-MT group relative to the HFD group (≥2 or ≤−2 fold, *P* < 0.05) (Fig. 2B). The DEGs between HFD and CD groups were primarily enriched in ECM-related pathways, such as collagen biosynthesis and enzyme modification. In contrast, DEGs between the HFD and HFD + 1-MT groups were mainly enriched in pathways related to neurotoxicity, such as neurexins and neuroligins (Fig. S2A, B). To better understand the antidepressant mechanism of 1-MT, we conducted pathway enrichment analysis on the shared DEGs between the CD vs HFD and HFD vs HFD + 1-MT groups. These genes represent those altered by HFD and further modified by 1-MT treatment, providing insight into the targeted pathways affected by 1-MT in the HFD model. Pathway analysis and network enrichment analysis of 130 shared differential genes showed that these DEGs mainly belong to developmentally relevant “embryonic pattern specification”, “pattern specification process” and “vesicle organization” pathways (Fig. 2C, D). These changes may correspond to abnormal neuroplasticity in the hippocampus, indicating that HFD has a significant impact on hippocampus function. Also, enrichment in immune response and autophagy-related pathways suggested neuronal damage and death involvement in the HFD-induced depression-like behavior and the therapeutic effect of 1-MT in reversing such phenotype (Fig. 2D). Importantly, we found a significant enrichment of DEGs in the ECM organization pathway. We cross-referenced our data with a curated dataset of matrix-associated genes [30] and identified nine genes encoding ECM-associated proteins, all of which showed a reverse effect upon 1-MT treatment (Fig. 2E). Among them, changes in Lgals3 and Col6a5 genes, whose expression results were also confirmed by qPCR (Fig. S2C, D), could affect the expression of chondroitin sulfate proteoglycans (CSPGs), the main component of PNNs [31, 32]. PNNs are ECM assemblies that preferentially unsheathe PV-positive interneurons, the loss of which has been found in major depressive disorder [33]. Since PNNs and PV-positive interneurons are known to modulate the development and plasticity of the CNS, we speculated that changes in the PNNs and PV-positive interneurons may be a key mechanism in the therapeutic effects of 1-MT.

### 1-MT treatment reverses the excess production of PNNs in HFD-induced depression

**Fig. 3:**
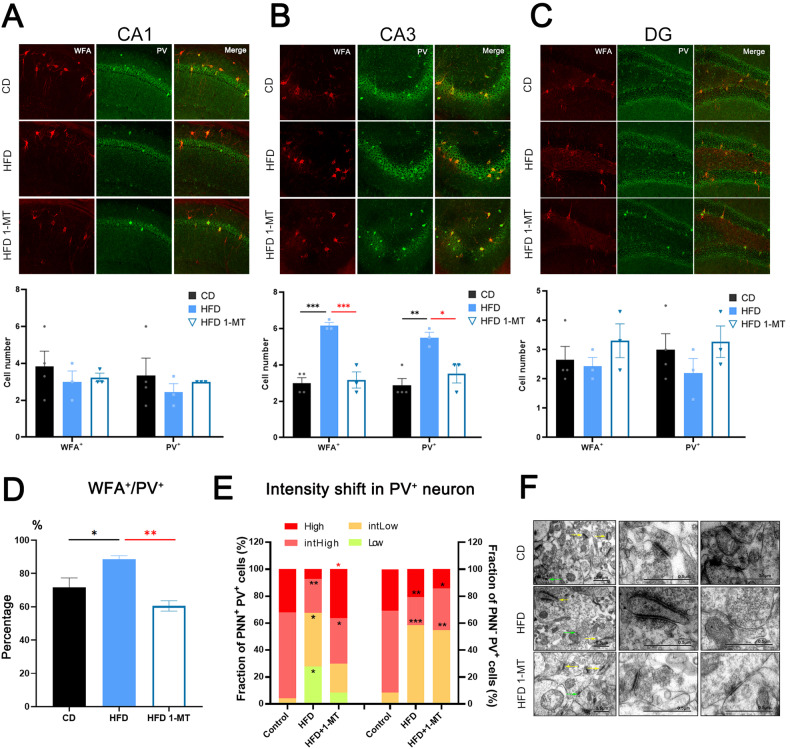
HFD-induced changes in PNNs and PV-positive neurons in the hippocampus, which are reversible by 1-MT. **A**–**C** WFA staining of PNNs in the CA3, CA1, and DG regions of the hippocampus in the CD and HFD groups, and the HFD group treated with 1-MT. Left panel, WFA middle panel, PV right panel, merge. **D** Quantification of the percentage of WFA-positive cells in PV-positive neurons of the CD and HFD groups and the HFD group treated with 1-MT. **E** Distribution of cells with different PV expression levels in PNN-positive (WFA labeled) and PNN-negative (WFA unlabeled) cells. **F** Representative electron micrographs showing examples of asymmetrical (yellow arrows) and symmetrical (green arrows) synapses onto neurons. Left panel, overview; middle panel, close-up of asymmetrical synapses; right panel, close-up of symmetrical synapses. Error bars represent the standard error of the mean. *P < 0.05, **P < 0.01, ***P < 0.001, as determined by the one-way ANOVA in combination with Tukey’s multiple comparisons test (**A**–**D**) or two-way ANOVA (**E**). *N* numbers are indicated in each graph. 1-MT 1-methyltryptophan, CD chow diet, HFD high-fat diet, PV parvalbumin, WFA wisteria floribunda agglutinin.

To investigate the therapeutic mechanism of 1-MT in HFD-induced depression-like behaviors, we first visualized PNNs in the hippocampal region using wisteria floribunda agglutinin (WFA) staining (Fig. 3A–C). WFA is a lectin that binds specifically to PNNs and is commonly used to label PNNs. We found that the number of PNN-coated neurons in the CA3 region of the hippocampus was increased in HFD-fed mice, but not in the CA1 or DG regions (Fig. 3A–C). Since PNNs mainly coat PV-positive neurons, we further quantified the number of PV-positive neurons in mice from both CD and HFD groups to clarify whether the increased number of PNNs is due to the increased number of PV-positive neurons, and found more PV-positive neurons in the HFD group. We also observed that the percentage of PNN-coated cells in PV-positive neurons increased in the HFD group. The data suggest that the increase in PNN-coated neurons was not solely due to an increase in the number of PV-positive neurons, but rather to more PV-positive neurons being coated by PNNs (Fig. 3D). Additionally, we observed a shift in PV expression levels, with an increase in cells with low and intermediate-low PV expression and a decrease in cells with intermediate-high and high PV expression in the HFD group (Fig. 3E). This intensity shift was also observed in PV-positive neurons that were not PNN-coated.

After the administration of 1-MT, the HFD-induced alteration of the ECM was reversed in the CA3 region of the hippocampus, as shown by the decreased number of PNN-coated neurons in the HFD + 1-MT group (Fig. 3A–C). 1-MT treatment can reduce the increased percentage of PNN-coated PV-positive neurons observed in the HFD group. Interestingly, administration of 1-MT can only partially rescue the intensity shift in PNN-positive interneurons, but not in PNN-negative cells (Fig. 3E). These findings suggest a correlation between the number of PV-positive neurons and PV expression with HFD and indicate that 1-MT may reverse HFD-induced depression-like behaviors through regulating PNNs.

PNNs have also been shown to play a regulatory role in synaptic plasticity. Synaptic plasticity is crucial for brain function, and it is thought to be disrupted in mood disorders such as depression. Increased PV immunostaining has been linked to reduced structural synaptic plasticity in the hippocampus [34]. Therefore, we analyzed the density of an active zone-associated protein Bassoon-positive synaptic puncta along the cell bodies of PNN-coated PV-positive interneurons in the CA3 region. We found increased Bassoon expression in PNN-free PV-positive neurons compared to their PNN-coated counterparts in both HFD and CD groups (Fig. S1L). However, no difference between the HFD and CD groups was observed. Overall, PV-positive neurons without PNN coating received more excitatory inputs than those with PNN coating, which was reflected by an increase in bassoon-positive puncta.

PV-positive neurons are the major type of GABAergic inhibitory interneurons in the hippocampus, and changes in their synapses may cause an excitation-inhibition imbalance in this region. To further clarify the involvement of neuronal synapses of PV-positive neurons in the therapeutic effect of 1-MT, we observed the ultrastructure of neuronal synapses by TEM and found that HFD induced a general increase in the length of excitatory synapses and a shortening of inhibitory synapses in the hippocampus compared to CD, and treatment with 1-MT reversed this change (Fig. 3F). Together, these data suggest that the therapeutic effect of 1-MT is through reversing the changes in the excitability of PV-positive interneurons in HFD-induced depression.

### 1-MT treatment reverses glutamate-GABA imbalance in the hippocampus of HFD-fed mice

**Fig. 4:**
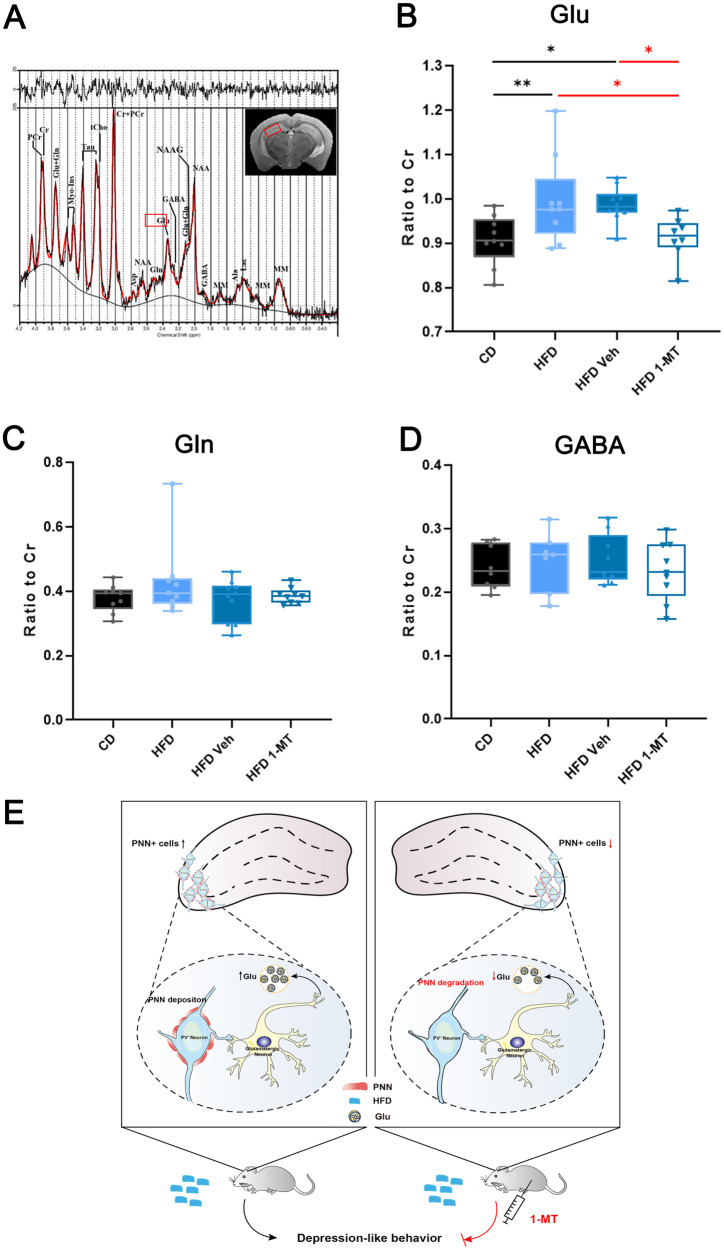
Effects of HFD and 1-MT administration on brain metabolites in the hippocampus. **A** Representative ^1^H MRS spectra from the hippocampus of HFD and CD mice. **B**–**D** Quantification of **B** glutamate, **C** glutamine, and **D** GABA levels in the hippocampus of CD and HFD mice, with or without 1-MT treatment. **E** A schematic diagram summarizing the proposed mechanism by which a long-term HFD induces depression-like behaviors through the excessive accumulation of PNN around PV-positive neurons, affecting synaptic transmission in PV-positive neurons, and leading to glutamate-GABA imbalance in the hippocampus. Error bars represent the standard error of the mean. *P < 0.05, **P< 0.01, as determined by the one-way ANOVA in combination with Tukey’s multiple comparisons test. *N* numbers are indicated in each graph. 1-MT 1-methyltryptophan, CD chow diet, GABA gamma-aminobutyric acid, Gln glutamine, Glu glutamate, HFD high-fat diet, PNN perineuronal net, PV parvalbumin.

Glutamate is the primary excitatory neurotransmitter in the brain, and glutamatergic mechanisms play key roles in the onset and development of depression. Lower glutamine and glutamate levels have also been found in the cortex of patients with depression [35]. We used ^1^H MRS, a noninvasive way to measure regional concentrations of brain metabolites, to quantify the effect of HFD and 1-MT treatment on neurotransmitters (Fig. 4A). The results showed that the level of glutamate, but not glutamine, was significantly higher in the HFD group compared to the CD group (Fig. 4B, C). The levels of GABA and other metabolite changes in the hippocampus associated with neural damage were not significantly different between the HFD and CD groups (Figs. 4D and S3A–F). 1-MT administration significantly reduced glutamate levels, suggesting its inhibitory effect on hippocampal hyperexcitability (Fig. 4B).

Taken together, we established a novel, PNN-related mechanism of 1-MT in reversing the HFD-induced depression, which involves reversing excessive accumulation of PNNs around PV-positive neurons and in turn affecting synaptic transmission in PV-positive neurons, leading to glutamate-GABA imbalance in the hippocampus (Fig. 4E).

## Discussion

Consistent with previous studies [36], we found that HFD can induce depression-like behaviors in mice. Additionally, we observed a reduction in hippocampal volume following a 23-week HFD consumption. Given the hippocampus’s critical roles in learning and memory [37, 38], and potentially endoreception [39], dietary habits like a western-style diet were implicated in impaired hippocampal-dependent learning and memory across various age groups, from prepubescent children [40] to older adults [41]. A diet high in saturated fat impairs hippocampal function by disrupting synaptic plasticity and neurogenesis in the hippocampus [42]. Impaired hippocampal function is often accompanied by changes in hippocampal volume. In vivo studies have demonstrated that HFD consumption during adolescence leads to a reduction in hippocampal volume [43]. The reduced hippocampal volume in HFD-fed mice in this study aligns with this notion and signifies impaired hippocampal-dependent emotion regulation, potentially contributing to the depression-like behavior observed.

Our findings highlight the extensive involvement in HFD-induced changes in hippocampal brain regions following HFD intake, as indicated by the significant enrichment of DEGs in the ECM organization pathways between the CD and HFD groups. Particularly, we identified the formation and accumulation of PNNs as a key change in this process. We conclude that long-term HFD consumption induces changes in ECM reorganization, namely the excessive accumulation of PNNs around PV-positive neurons, which in turn affects synaptic transmission in PV-positive neurons and leads to glutamate-GABA imbalance in the hippocampus. To our knowledge, the role of the hippocampal ECM in HFD-induced depression-like behaviors has not been studied previously. Our study addresses this gap in the literature and sheds light on the contribution of hippocampal ECM reorganization to the depressive phenotypes associated with HFD.

Aberrant PNNs have been reported to be associated with neurodegenerative and neuropsychiatric disorders through abnormal neuroplasticity [44–48]. However, little is known about the effect of HFD on PNNs in hippocampal brain regions. We found that HFD led to an increase in PNNs and PV-positive neurons in the hippocampal CA3 region. PNN-coated PV-positive neurons are widely distributed and abundant GABAergic inhibitory interneurons that provide local feedforward and feedback inhibition through perisomatic boutons onto principal excitatory neurons [49–51]. The reduced PV levels observed in PV-positive interneurons indicate the level of GABA-producing GAD-67, suggesting a diet-related reduction in overall GABA-mediated hippocampal inhibition [52]. We also observed that HFD resulted in a general increase in the length of excitatory synapses and a shortening of inhibitory synapses. Further evidence demonstrated an increase in the level of glutamate in the hippocampal region of HFD-fed mice, and this increase may be due to a weakening of the inhibitory capacity of PV-positive neurons.

In the present study, significant reductions in the hippocampus 5-HT level, 5-HT/Trp ratio, and elevated 5-HIAA/5- HT ratio were observed in HFD-fed mice, indicating that the depression-like behavior might be attributed to serotonin turnover. This is consistent with the elevated blood serotonin turnover observed in patients with depression, with previous studies supporting the role of 5-HT in mood regulation and depression [28, 29]. The levels of Trp and Kyn showed no changes, suggesting that the neurotoxic mechanisms mediated by the Kyn pathway and its metabolites may not be involved in the development of the pathological state in our model. To our surprise, 1-MT significantly increased the levels of 5-HT. Previous studies have demonstrated that medications targeting 5-HT can reduce energy intake and reverse weight gain in HFD-fed rats [53]. While our data did not show a reduction in body weight or adipose tissue mass with 1-MT treatment, we observed a significant improvement in glucose tolerance in HFD-fed mice. Obesity leads to adipose tissue remodeling, and notably, the expansion of visceral WAT is strongly associated with the development of insulin resistance [54]. This insulin resistance, compounded by obesity-induced inflammation, can disrupt insulin signaling, ultimately leading to glucose intolerance and metabolic disorders. In addition, it has been shown that 5-HT can regulate insulin secretory function in beta cells via the 5-HT3 receptor under HFD-induced metabolic stress conditions [55], which may also be a potential mechanism for 1-MT treatment to rescue the HFD-induced impaired glucose tolerance.

The therapeutic role of 1-MT in depression has been widely demonstrated, but its role in obesity-induced mood disorders remains unclear. Conventionally, 1-MT is known to exert its antidepressant effect through competitively inhibiting IDO activity [56–58]. However, in the present study, 1-MT reversed depression-like behaviors without altering IDO activity in HFD-fed mice. A number of previous studies have confirmed that 1-MT has little to no effect on IDO activity in control mice, and the inhibition of IDO by 1-MT is not consistently effective [56, 59]. It has been demonstrated that 1-MT interferes with TLR signaling in dendritic cells independently of IDO activity. Additionally, IDO activity and Kyn levels remained unchanged after 2 weeks of continuous administration of Trp analogs, which may reflect the TDO-dependent degradation of Trp into Kyn to maintain Kyn levels [60]. These findings suggest that the antidepressant effects of 1-MT may involve alternative mechanisms unrelated to IDO activity.

Interestingly, we found that 1-MT treatment reversed the increase in PNNs. Chronic administration of selective 5-HT reuptake inhibitors has been found to impact PNN deposition around PV interneurons and is implicated in fear erasure in the basolateral amygdala [61] and in the reopening of critical period plasticity in the visual cortex, likely through the dissolution of PNNs in adulthood [62]. Here, we hypothesize that the degradation effect of 1-MT on PNNs may involve the regulation of 5-HT. Serotonin is known to modulate neuronal morphology and dendritic spine shape and density [63–65]. Developmental knockout of the 5-HT transporter is associated with increased dendritic complexity in infralimbic cortex pyramidal neurons and increased spine density in basolateral amygdala pyramidal neurons [66]. Additionally, 5-HT can affect neuronal function by influencing synaptic plasticity. Recent studies have shown substantial decreases in GABA-Aα2 receptor subunits in the hippocampus of animals treated with fluoxetine, as well as increased neuronal activity [22]. Considering the impact of 5-HT treatment on PNN expression, it is reasonable to suspect that there may be a pathway in the regulation of mood by 5-HT that disrupts hippocampal excitation-to-inhibition homeostasis by affecting PNN expression and ultimately leads to the development of depression-like behaviors. The results supporting this hypothesis were that 1-MT treatment improved 5-HT expression, normalized PNN expression, and subsequently restored the excitation-to-inhibition imbalance in the hippocampus, thereby ameliorating depression-like behaviors.

Reduced hippocampal volume serves as a biomarker for identifying severe depression and reflects the long-term effects of various unknown stresses, as evidenced by reduced hippocampal volume in drug-free depressed patients [67]. In our study, we observed a decrease in hippocampal volume after 23 weeks of HFD intake, indicating the neurotoxic effects of HFD on the brain. However, we did not observe significant changes in hippocampal volume in the HFD group after 1-MT treatment. This could be because the duration of the 1-MT administration was too short to reverse the structural changes. Supporting this hypothesis, we have observed the therapeutic effect of 1-MT on synaptic plasticity in the hippocampus.

The present study is subject to several limitations. Firstly, the optimal behavioral assessments for measuring depressive phenotypes in rodent models are a subject of ongoing debate [68]. Although FST was originally developed to evaluate the effectiveness of antidepressant treatments, recent studies have questioned its utility in assessing depression induction [69]. Secondly, gender disparities in clinical depression are well documented [70]. Consequently, it is plausible that our observations in male mice may not be wholly applicable to female mice.

In conclusion, our study shows that 1-MT treatment can reverse HFD-induced depression-like behaviors in mice by reversing HFD-induced ECM remodeling. HFD induces depression-like behavior by promoting ECM remodeling in the hippocampus, including excessive accumulation of PNNs around PV-positive neurons, which results in synaptic abnormalities and induces glutamate-GABA imbalance in the hippocampus. The discovery that 1-MT can reverse such changes in the hippocampus provides a potential novel therapeutic option for patients with diet-induced depression.

### Supplementary information

Supplemental figures with captions

Supplemental Table 1

## Data Availability

All data are available in the main text or the supplementary materials except for RNA-sequencing data and MRI images. RNA-sequencing data has been deposited and MRI images will be made available on request.
