# Supplementary material for: 1-Methyltryptophan treatment ameliorates high-fat diet-induced depression in mice through reversing changes in perineuronal nets
Source: Transl Psychiatry. 2024 May 30;14:228. doi: 10.1038/s41398-024-02938-4 (PMC11139877; doi:10.1038/s41398-024-02938-4)
Supplement: Supplementary file 1 — Supplemental figures with captions [file 41398_2024_2938_MOESM1_ESM.docx]

Supplementary Materials for

1-methyltryptophan treatment ameliorates high-fat diet induced depression in mice through reversing perineuronal nets changes

**
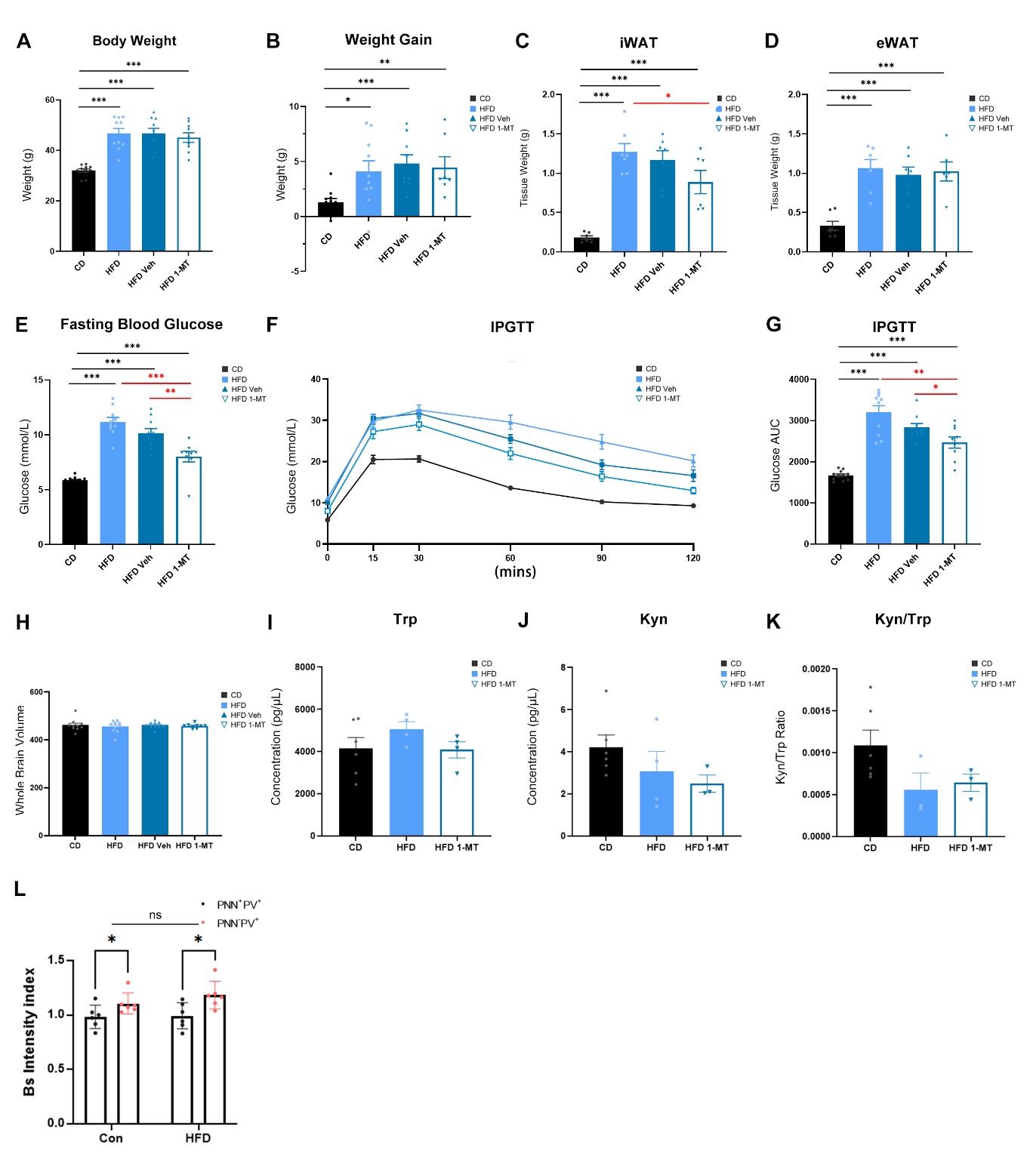
**

Fig. S1 (A to E) Establishment of an HFD-induced obesity mouse model with mice fed with HFD or standard CD for 18 weeks. (A) Body weight, (B) weight gain, (C) inguinal white adipose tissue (iWAT) weight, (D) epididymal white adipose tissue (eWAT) weight, (E) results of the glucose tolerance test (GTT), and (F to G) the intraperitoneal intraperitoneal glucose tolerance test (IPGTT) of mice fed with CD,

HFD, HFD treated with vehicle, and HFD treated with 1-MT. (H) Quantification of the whole brain volume of mice fed with CD, HFD, HFD treated with vehicle, and HFD treated with 1-MT. (I to K) shows the tryptophan (Trp), kynurenine (Kyn), and Trp/Kyn ratios in the hippocampus of mice fed with CD, HFD, and HFD treated with 1-MT. (L) Bassoon expression in PNN-free PV-positive and PNN-coated neurons counterparts in CD and HFD groups. Data are presented as mean ± SEM. Statistical significance is indicated by *p < 0.05, **p < 0.01, and ***p < 0.001.


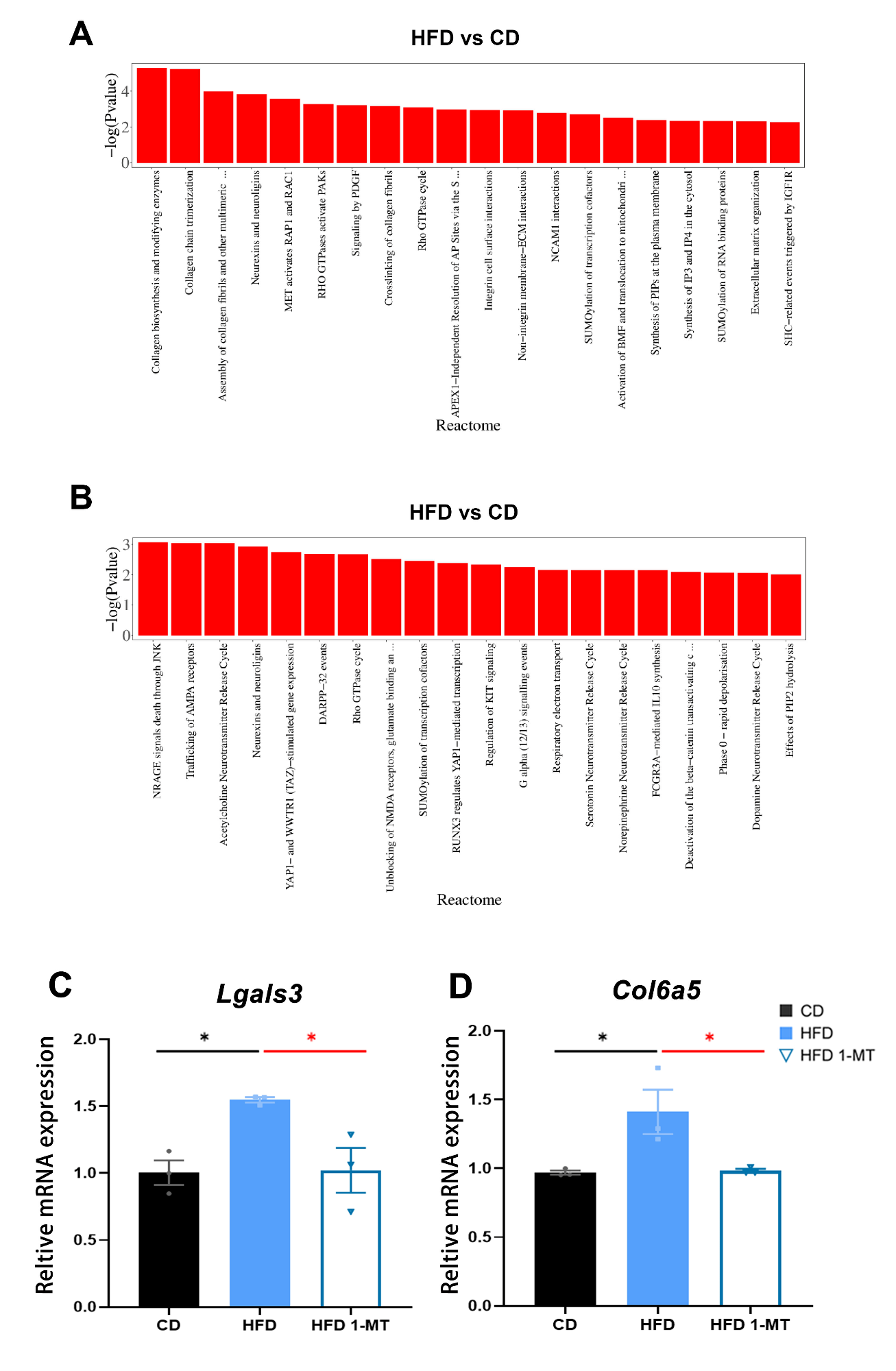


**Fig. S2 Pathway analysis of differentially expressed genes in the hippocampal expressional profiles of mice in the CD group, HFD group and HFD with 1-MT treatment and qPCR results of two signature ECM genes.** Data are presented as mean ± SEM. N numbers are indicated in the figure. Statistical significance is indicated by *P < 0.05.


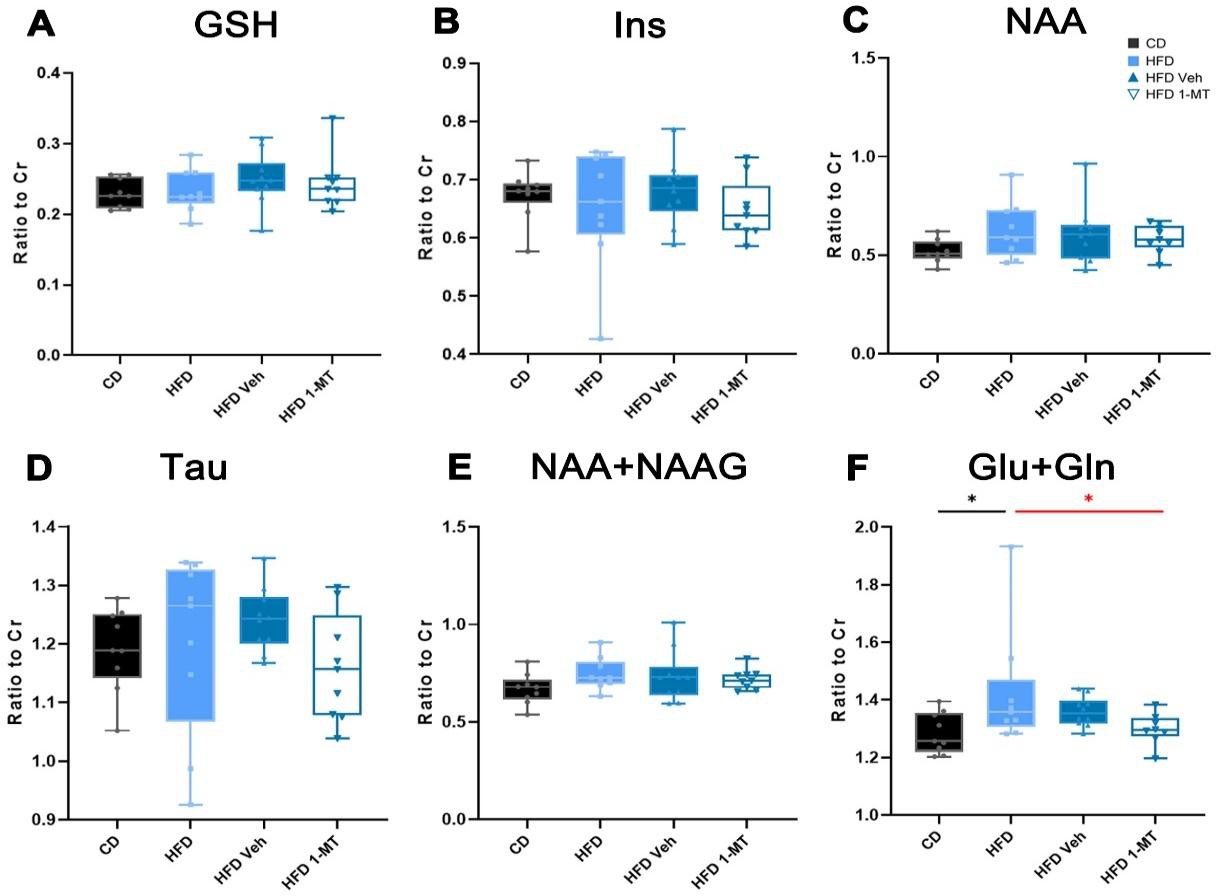


# Fig. S3 Metabolite changes in the hippocampus associated with neural damage in mice fed with CD, HFD, HFD treated with vehicle, and HFD treated with 1-MT.

(A) Glutathione (GSH). (B) Myo-inositol (Ins). (C) N-acetylaspartate (NAA). (D) Tau proteins. (E) N-NAA and Acetylaspartylglutamic acid (NAAG). (F) Glutamate (Glu) and glutamine (Gln). Data are presented as mean ± SEM. N numbers are indicated in the figure. Statistical significance is indicated by *P < 0.05.

# MATERIALS AND METHODS

**Behavioral tests**

Tail suspension test (TST)

Mice were individually taken from their home cage and suspended by tape from the tip 1/3 of their tails. The head was at the same level as the camera, about 5 cm from the bottom. After the first 2 minutes of acclimatization, the animal immobility time (recognized as frustration and a lack of active struggle for survival) was recorded during the last 4 minutes by a video tracking system.

Forced swimming test (FST)

Mice were taken from their home cage and then separately put into a cylindrical glass container (100 mm diameter, 300 mm height) with 200 mm and 23 ± 1°C water, and forced to swim for 4 minutes after a 2-minute habituation period with their nose kept above the water level. The immobility time (identified as when the mouse ceased to struggle and floated in the water) was recorded by a video-tracking system. After the test, the mouse was retrieved from the water, immediately dried and returned to its home cage.

Open field test (OFT)

Mice were individually placed in the center of the open field (100cm×100cm×40cm) in a brightly lit room, and allowed free and uninterrupted movement for 5 minutes. Following each test, the site was deodorized with alcohol and dried with towels. The distance of mouse movement and the time of mouse in the central and peripheral areas were recorded by video tracking software.

Elevated zero maze (EZM) test

Mice were individually placed in the middle of the intersection of the open and closed arms of the maze (inner circle diameter 290 mm, outer circle diameter 400 mm, inner and outer baffle height 155 mm, runway width 50 mm) with the head toward the center of the circle. During the test, mouse behavior was recorded for 5 minutes with a camera system. The time and number of mice that visited the open or closed quarters were recorded and analyzed.

Sucrose preference experiment (SPT)

The SPT was performed in two parts: adaptation and preference. During adaptation, mice were continuously exposed to two bottles containing 2% (wt/vol) sucrose solution for 24 hours. Immediately following the 24 hours, one of the bottles was replaced with fresh water. The placement of the two bottles was changed after 12 hours. At the end of acclimatization, mice were deprived of food and water for 24 h. During the preference test, each mouse was exposed to two bottles (one with 2%

[wt/vol] sucrose solution, the other with fresh water) for 24 hours. After 12 hours, the positions of the two bottles were exchanged. The consumption of sucrose solution and fresh water were measured respectively as follows: sucrose intake (g) × 100%/ (sucrose intake [g] + water intake [g]).

# RNA sequencing

The company used Trizol to extract total RNA from tissues, and used the double-end sequencing mode of the Illumina Hiseq sequencing platform to perform high-throughput sequencing of total RNA from hippocampal tissues in the CD, HFD, and HFD+1-MT groups, with three replicate samples in each group. FastQC software was used for quality control analysis of the pre-processed data as well as to count the base ratios of Q20 and Q30.

# Immunohistochemistry

Mice were anesthetized (4 ml/kg, 10% chloral hydrate), irrigated with PBS, and fixed with 4% paraformaldehyde. Fixed brains were separated and post-fixed in 4% paraformaldehyde at 4°C overnight, respectively, dehydrated with 20% or 30% sucrose solution (PBS as solvent), and blocked in the Tissue-Tek OCT compound at

-20°C. Brain tissue was sliced into sections at 40-μm thickness (CM1850; Leica Microsystems, Wetzlar, Germany) and put into an anti-freeze solution (glycerin: PBS

= 1[v]:1[v]). Slices were picked out of anti-freeze solution, immersed in PBST (0.1 % Triton X-100 [Beyotime] in PBS) for 10 min three times, permeabilized and blocked with 5% BSA (Sigma) for 2 h at room temperature, and exposed overnight to primary antibody mixtures: biotin-conjugated WFA (1:500, Sigma-Aldrich, L-1516), anti-parvalbumin antibody (1:500, Millipore, MAB1572) and anti-Bassoon antibody (1:500, Novus Biological, NBP1-46351) at 4 ℃ . Following three washes in PBST, slices were incubated for 2 h at room temperature with the appropriate secondary antibody conjugated with Alexa Fluor 647, Alexa Fluor 488, or Streptavidin-Alexa Fluor 568 (Thermo Fisher, S11226), diluted 1:500 in PBS-T, and then bound with DAPI to label the cell nuclei. Images were attained with a Zeiss confocal microscope (LSM 700).

# Magnetic resonance imaging scans and 1H-Magnetic Resonance Spectroscopy

Data acquisition

MRI experiments were conducted at a Bruker BioSpec 11.7T scanner, using 86 mm volume coil for transmission and 4 channel phased array cryogenic mouse head coil for receiving. Mice were mounted in an animal holder using a tooth bar and ear bars for stable positioning. Body core temperature was controlled and monitored with an in-house designed automated temperature control unit complete with water blanket. After behavioral tests, mice were anaesthetized with isoflurane (1% oxygen and 5% isoflurane at induction, 1% oxygen and 1% isoflurane during continuous anesthesia).

T2 RARE anatomical images acquired with axial scanning were used to estimate hippocampus volume after a localizer scan for animal positioning (TE = 30 ms, TR = 4500 ms, field of view = 18 × 18 mm2, matrix size = 256 × 256, slice thickness = 0.3 mm, number of slices = 43, average = 1). A T2 RARE anatomical image with sagittal scanning were acquired for MRS location (TE = 30 ms, TR = 1800 ms, field of view

= 20 × 18 mm2, matrix size = 256 × 256, slice thickness = 0.5 mm, number of slices = 17, average = 1). After field-map-based local shimming was performed within the mouse brain, for 1H-Magnetic Resonance Spectroscopy (1H-MRS), a point-resolved spectroscopy (PRESS) sequence was used. Field inhomogeneity was improved by shimming. Spectral quality was considered acceptable if the full width at half maximum (FWHM) was not larger than 20 Hz. The parameters used for 1H-MRS location were acquired with following parameters: TE = 16.2 ms, TR = 2500 ms,average = 256,voxel size = 1.7 × 1.2× 1.6 mm3,and acquisition time = 10 min 40s. For hypothalamus and hippocampus location, the voxel size was set at 2.2 × 1× 2 mm3 and 1.7 × 1.2× 1.6 mm3 respectively.

Data processing

The hippocampus region was manually delineated using ITK-snap, and the hippocampus volume was estimated from the segmentation.

1H-MRS spectra were processed using LC Model software (Version 6.3-1L), which analyzes an in vivo spectrum as a Linear Combination of Model in vitro spectra from individual metabolite solutions (Provencher 1993). Absolute metabolite levels were calculated, including creatine (Cr),phosphocreatine(PCr), myo-inositol(myo-Ins), γ-aminobutryric acid (GABA), glutamine (Gln), glutamate (Glu), glycerophoshocholine (GPC), glutathione (GSH), N-acetyl-aspartate (NAA), N-acetyl-aspartyl-glutamate (NAAG) , and taurine (Tau). Besides, total creatine was calculated as tCr from PCr and Cr. Metabolite concentrations were also given relative to tCr and taken as relative values (myo-Ins/tCr, GABA/tCr, Gln/tCr, Glu/tCr, GPC/tCr, GSH/tCr, NAA/tCr, Tau/tCr, Glu+Gln/tCr, and NAAG+NAA/tCr).

Manual segmentation of hippocampal brain regions and volumetric measurements

Apply ITK-SNAP (Version: 3.8.0) (1) software to draw the hippocampal area manually. ITK-SNAP provides a semi-automatic segmentation capability to analyze the selected area and obtain its 3D model. For each MRI image, we manually drew the hippocampal boundaries layer by layer according to the mice brain atlas to generate hippocampal labels. The volume of the hippocampus was obtained based on the labels corresponding to the hippocampal brain regions using the label volume calculation tool that comes with the software.

References

1. YUSHKEVICH P. A., PIVEN J., HAZLETT H. C., SMITH R. G., HO S., GEE J.

C. et al. User-guided 3D active contour segmentation of anatomical structures: significantly improved efficiency and reliability, Neuroimage 2006: 31: 1116-1128.
